# Operationalizing Competency-based Medical Education Within Clinical Competency Committees

**DOI:** 10.5811/westjem.53819

**Published:** 2026-05-13

**Authors:** Andrew Golden, Sara Dimeo, Caroline Molins, Paul Kukulski, Kaitlin Ray, Benjamin Schnapp, Laura Hopson

**Affiliations:** * Case Western Reserve University School of Medicine, University Hospital Cleveland Medical Center, Department of Emergency Medicine and Medical Education, Cleveland, Ohio; † Creighton University School of Medicine-Phoenix, Department of Emergency Medicine, Phoenix, Arizona; ‡ University of South Alabama, Department of Emergency Medicine, Mobile, Alabama; § University of Chicago, Department of Medicine, Chicago, Illinois; ¶ University of Michigan, Department of Emergency Medicine, Ann Arbor, Michigan; || Vanderbilt University Medical Center, Department of Emergency Medicine, Nashville, Tennessee; # University of Wisconsin School of Medicine and Public Health, Department of Emergency Medicine, Madison, Wisconsin

## Abstract

This scholarly perspective explores the integration of competency-based medical education (CBME) within graduate medical education assessment systems, specifically the clinical competency committee (CCC). We discuss the role of the CCC in operationalizing the core components of CBME, providing guidance on best practices-related meeting structure, assessment data, and learner outcomes. By analyzing the evolving responsibilities of faculty assessors and the impact on learner progression toward unsupervised practice, this perspective highlights challenges and strategies for successful implementation of CBME principles in medical education, including an outline to use when discussing each trainee in CCC meetings.

## INTRODUCTION

As graduate medical education adopts competency-based medical education (CBME), existing assessment systems must evolve to align with CBME principles. Competency-based medical education is “an approach to preparing physicians for practice that is fundamentally oriented to graduate outcome abilities and organized around competencies derived from an analysis of societal and patient needs.”^1^ It integrates five components: outcome competencies, sequenced progression, tailored learning experiences, competency-focused instruction, and programmatic assessment (Table).^2^ In this way, CBME uses a framework to guide individual learners and faculty assessors in holistic summative entrustment decision-making to drive learner progression in awarding new graduated responsibility up to and including unsupervised practice.

The group tasked with summative assessment of resident physicians in the United States is the clinical competency committee (CCC).^3^ The decisions made by these committees are crucial to ensuring physicians are competent to provide safe patient care. Despite calls for CCCs to develop standardized structures and functions, recommendations have not yet fully integrated the principles of CBME.^2^,^4^ Our goal in writing this scholarly perspective was to highlight strategies for CCCs to meaningfully integrate CBME principles. We draw on literature from both the health professions and other fields to inform practices centered around the themes of meeting structure and culture, data gathering, and learner-centered outcomes. We include the Box below as an example of the assessment data and recommendations that can be used in preparation for, during, and at the conclusion of a CCC meeting as a summary of this perspective.

Box **Example clinical competency committee data and recommendations**

Resident:

PGY:

Prior CCC Recommendations:

Review:

1. Previous milestone assignments
2. Knowledge assessments
  - In-training Exam
  - Question Bank Completion
3. Workplace-based Assessments
4. End-of-rotation Assessments
5. 360-degree Assessments
  - Peers
  - Patients
  - Interprofessional Team Members
6. Administrative data
  - Attendance at required didactics
  - Procedure/Case Log Completion
  - Quality Improvement Referral Metrics

Current CCC Recommendations

Based on the data analyzed during this meeting, this resident should receive (circle one):

**Table:** 

Promotion	Personal Improvement Plan	Probation	Termination

Specific Guidance using Start, Stop, Continue Framework (examples provided)

1. Stop anchoring on diagnoses. Consider broadening or altering your differential and changing patient disposition when needed as you interpret results of labs, imaging, and response to therapeutics.
2. Start incorporating closed-loop communication during resuscitations. Use names, when possible, to avoid confusion and ensure each team member understands which tasks to address.
3. Continue your excellent documentation. Your assessments and plans are clear and concise, allowing all members of the team to understand the workup and care of your patients.

*CCC*, clinical competence committee*; PGY*, postgraduate year.

## PART I: MEETING STRUCTURE AND CULTURE

### Developmental Model

A core tenet of the CCC is to maintain a meeting culture, group members, and processes that allow for effective review of each individual learner’s progression to make summative entrustment decisions. Rather than a “problem-focused” model that primarily targets struggling learners, the adoption of a developmental model facilitates discussions centered on well-defined benchmarks. By adopting a developmental paradigm rather than one focused solely on deficiencies, CCCs are directed toward competency-based outcomes for all learners.^5^

#### Group Members

Members of the CCC should represent a range of backgrounds, academic ranks, and expertise. This diversity lessens homogenous decision-making by incorporating a spectrum of perspectives and is a key component to increasing equity in assessment.^6^,^7^ Practices such as encouraging participation from junior members before senior members may promote healthy debate, allow for respectful questioning, and aid in reaching well-informed decisions.^6^,^8^ Avoiding “groupthink,” or conforming to an idea without meaningful discussion or challenge, is essential to fostering meaningful dialogue among CCC members.^9^ Assigning a CCC member to be a strategic dissenter (“devil’s advocate”) can help pinpoint problem areas, identify patterns, and improve the quality of the group’s decision-making.^10^ This individual can also be tasked with identifying and responding to groupthink.

#### Meeting Structure

Clinical competency committees are required to meet at least twice annually, which can generate time pressures in striving to discuss each resident holistically.^3^,^11^ CCCs, particularly those within larger programs, may consider meeting more regularly (ie, quarterly or monthly) or designating meetings by postgraduate year. This generates more timely and frequent feedback to trainees while also allowing CCCs to identify patterns of performance.^8^

Each meeting of the CCC should begin with a “mission moment” to remind members of their purpose in guiding learners toward competence and cultivating a culture of growth.^8^,^12^ When discussing trainees, CCC members should maintain a constructive and respectful tone that encourages open dialogue and idea exchange. It is critical that CCCs base dialogue in assessment data, while understanding that compiled data may be incomplete or contradictory.^13^ The resulting group discussion should focus on reconciling conflicting information and sharing a collective responsibility for decision-making. In this way, the CCC fulfills its role to support program directors in justifying or enforcing decisions.^5^

Finally, CCCs should regularly engage in evaluative practices and continuous quality improvement to ensure that the committee’s values, processes, and systems remain aligned with their mission of developing trainee competence. This includes gathering CCC member feedback on meeting structure, data analysis methods, and communication strategies. Regular reflection and adaptation can enhance the committee’s effectiveness and ensure that meetings remain productive and conducive to achieving CBME principles within the CCC.^8^ Ongoing faculty development around CBME can assist in maintaining fair assessment processes.^14^

## PART II: DATA GATHERING

A crucial component of CBME is the collection and collation of timely and accurate trainee data to inform discussions during CCC meetings. Given the CCC’s role in high-stakes progression decisions, robust data collected from a variety of formats, perspectives, and contexts within a clear organizing framework are essential. This principle, also known as programmatic assessment, offers the opportunity to map data sources within a residency program to provide an overview of how and by what methods each learner is assessed.^15^,^16^

We describe several methods through which programs can integrate CBME principles rooted in programmatic assessment to assist CCCs in their work. Programs should gather frequent assessments of learners so that data can be organized and processed continuously and longitudinally across contexts rather than at singular points in time.^17^ Furthermore, assessments should include quantitative and qualitative data to generate a holistic picture of resident performance.^4^ These individual assessments should be mapped to specific competencies and/or entrustable professional activities (EPA), ensuring no domains of competence are under-assessed.^18^ Finally, programs should define acceptable performance standards and communicate these with trainees.

The EPAs are an assessment framework well-suited for CMBE. These are “units of professional practice that can be fully entrusted to a trainee, as soon as he or she has demonstrated the necessary competence to execute this activity unsupervised.”^19^ When integrated into workplace-based assessments, EPA frameworks are easy to assess by clinical faculty, longitudinally track a trainee’s progress toward independent practice, and easily map to Accreditation Council of Graduate Medical Education (ACGME) subcompetencies for reporting.^19^–^22^ Twenty-two EPAs have been developed within emergency medicine (EM).^23^ These are currently being piloted at a number of EM residency training programs across the United States.^24^

Traditional methods of assessment include tests with multiple-choice question and formative clinical assessments. As programs transition to CBME, additional attention should be placed on gathering assessment data in supplemental methods, such as through simulation activities, procedural assessment, direct observation on shift, and multisource feedback (ie, patients, peers, and other healthcare team members). Integrating these practices can create a comprehensive snapshot of learner performance while also contributing to an assessment culture focused on equity.^25^,^26^

## PART III: LEARNER-CENTERED OUTCOMES

After CCC meetings, the committee’s discussion and interpretation of data must be translated into learner-centered outcomes and delivered to trainees in a way that promotes their development. The goal of the CCC should be to reinforce positive behaviors and modify negative behaviors.^27^ By acknowledging exemplary behaviors, trainees may be motivated to repeat them, resulting in increased confidence in their skills.^28^ Equally important, the CCC must identify and provide specific guidance to trainees as to how they can modify negative behaviors and performance.^28^

One strategy educators may employ to communicate CCC recommendations includes the “Stop, Start, Continue” approach.^27^ These outline which behaviors to stop, which to start, and which to continue on a trajectory toward independent practice.^27^ In generating these recommendations, educators should ensure statements are specific, actionable, focused on growth, and examined for bias.^27^

Providing learner-centered outcomes for each trainee after CCC meetings is an integral way programs can transition toward CBME. When communicating recommendations for trainees, written summaries accompanied by discussion from a trusted source may be the most beneficial.^5^,^27^ Beginning performance conversations with self-assessments also allows trainees to reflect on their performance, thus facilitating corrective feedback to feel more acceptable and instructive.^28^

## CONCLUSION

This scholarly perspective offers practical guidance for programs to integrate the principles of competency-based medical education into their clinical competency committees. By integrating these principles, program and CCC leaders can work to ensure trainees are fairly and equitably assessed on their path to independent practice.

## Figures and Tables

**Table:** The five components of competency-based medical education and their Integration into the clinical competency committee.^2^

CBME Core Component	Definition	CCC Role in Competency
Outcome Competencies	“Competencies required for practice are clearly articulated.”^2^	Utilize established frameworks to define competency, such as (1) ACGME competencies and subcompetencies or (2) EPAs, and communicate these to learners.
Sequenced Progression	“Competencies and their developmental markers are sequenced progressively.”^2^	Recognize progression toward independent practice as defined by established frameworks, such as (1) ACGME Milestones or (2) EPAs. In assessing this progression, acknowledge learners will move along different trajectories.
Tailored learning experiences	“Learning experiences facilitate the developmental acquisition of competencies.”^2^	Identify individual resident deficiencies with recommendations that may include additional exposure to or remediation of specific rotations. Systemic lapses in progression toward independent practice should be referred for evaluation by residency leaders and/or the Program Evaluation Committee.
Competency-focused instruction	“Teaching practices promote the developmental acquisition of competencies.”^2^	Provide CCC recommendations that can be turned into goals and implementation plans with program leaders, trainees, and/or coaches.
Programmatic assessment	“Assessment practices support and document the developmental acquisition of competencies.”^2^	Ground CCC conversations about resident progression in robust assessment data. Inadequate data from faculty or residents should be referred to program leaders and/or the Program Evaluation Committee for further development.

*ACGME*, Accreditation Council for Graduate Medical Education; *CBME*, competency-based medical education; *CCC*, clinical competency committee; *EPAs*, entrustable professional activity.
